# Nonwoven Reinforced Photocurable Poly(glycerol sebacate)-Based Hydrogels

**DOI:** 10.3390/polym16070869

**Published:** 2024-03-22

**Authors:** Michael Phillips, Giuseppe Tronci, Christopher M. Pask, Stephen J. Russell

**Affiliations:** 1 Clothworkers’ Centre for Textile Materials Innovation for Healthcare, Leeds Institute of Textiles & Colour, School of Design, University of Leeds, Leeds LS2 9JT, UKg.tronci@leeds.ac.uk (G.T.); 2 School of Chemistry, University of Leeds, Leeds LS2 9JT, UK

**Keywords:** polymer, hydrogel, elastomeric, composite, fibre-reinforced, photocurable, photoresponsive, nanofibre, electrospun, UV-cured, nonwoven, regenerative medicine, structural support scaffold, tissue engineering, biomaterial

## Abstract

Implantable hydrogels should ideally possess mechanical properties matched to the surrounding tissues to enable adequate mechanical function while regeneration occurs. This can be challenging, especially when degradable systems with a high water content and hydrolysable chemical bonds are required in anatomical sites under constant mechanical stimulation, e.g., a foot ulcer cavity. In these circumstances, the design of hydrogel composites is a promising strategy for providing controlled structural features and macroscopic properties over time. To explore this strategy, the synthesis of a new photocurable elastomeric polymer, poly(glycerol-*co*-sebacic acid-*co*-lactic acid-*co*-polyethylene glycol) acrylate (PGSLPA), is investigated, along with its processing into UV-cured hydrogels, electrospun nonwovens and fibre-reinforced variants, without the need for a high temperature curing step or the use of hazardous solvents. The mechanical properties of bioresorbable PGSLPA hydrogels were studied with and without electrospun nonwoven reinforcement and with varied layered configurations, aiming to determine the effects of the microstructure on the bulk compressive strength and elasticity. The nonwoven reinforced PGSLPA hydrogels exhibited a 60% increase in compressive strength and an 80% increase in elastic moduli compared to the fibre-free PGSLPA samples. The mechanical properties of the fibre-reinforced hydrogels could also be modulated by altering the layering arrangement of the nonwoven and hydrogel phase. The nanofibre-reinforced PGSLPA hydrogels also exhibited good elastic recovery, as evidenced by the hysteresis in compression fatigue stress–strain evaluations showing a return to the original dimensions.

## 1. Introduction

Hydrogels are widely applied to support the repair and regeneration of soft tissues, though their large water content can limit their clinical handling, fixability to surrounding tissues and wet state mechanical properties. To overcome these issues, non-hydrophilic alternatives may be preferred over systems with enhanced regenerative functionality. One example is in the clinical treatment of ulcerative chronic wounds of the lower feet, specifically cavities caused by tissue loss in the plantar fat pad. Currently, repeated silicone injections can be used to fill such cavities to restore a degree of mechanical function, but the treatment provides no regenerative capacity. In situations such as this, the ability to switch to a bioresorbable elastomeric hydrogel, with appropriate load-bearing properties, could provide scope for both regenerative as well as mechanical functions.

In seeking suitable injectable alternatives, poly(glycerol sebacate) (PGS) is an elastomeric, bioresorbable polymer that has been previously deployed in a variety of biomedical applications and possesses a valuable combination of physical and biological properties. Harding et al. [1] produced PGS-based patches for the delivery of embryonic stem cells to the heart, demonstrating that cardiomyocytes remain active on the plate for longer than three months until interrupted. PGS has also been investigated for elastomeric scaffolds used for small-diameter bypass grafts, and potential advantages have been identified in relation to elastin expression [2]. Scaffolds containing PGS are reported to promote proliferation and phenotypic protein expression in relation to vascular cells [3]. PGS-based materials therefore offer the potential to develop biocompatible elastomeric materials, combined with a regenerative function for the repair and regeneration of soft tissues.

To further improve the bulk mechanical properties of PGS, particularly when delivered in the form of a hydrogel, micro- or nanofibre reinforcement can be harnessed. Nanofibrous nonwovens are engineered fibrous assemblies containing fibres with diameters up to ~500 nm [4]. Previously, microfibre-reinforced hydrogel scaffolds have been produced by melt-electrowriting (MEW) processes, among other methods [5]. Huang et al. demonstrated that the integration of nanofibres into an alginate-based hydrogel yielded mechanical behaviour consistent with native tissue, with the compressive stress testing revealing a ‘J-curve’ response [6]. The stress increased by 87% with a 30% nanofibre content. Electrospun gelatin nanofibres have also been used to reinforce alginate hydrogels in scaffolds for corneal tissue engineering, with mechanical properties an order of magnitude higher than for the hydrogel materials alone [7]. Martin and Youssef [8] also studied the dynamic properties of hydrogels with respect to load bearing for biomedical applications.

Previous work on the production of PGS nanofibres has shown promise for tissue engineering purposes. Gultekinoglu et al. [9] blended a PGS prepolymer with poly(vinyl alcohol) (PVOH); blending the un-crosslinked PGS prepolymer with PVOH avoids the solubility and melting issues associated with preparing electrospinning solutions of crosslinked PGS. The resulting fibres had a uniform size distribution, which was maintained even after thermal crosslinking. After washing to remove PVOH, the average fibre diameter decreased by approximately 25%. The study characterised the chemical structure, morphology, and cell viability of the PGS fibres, demonstrating that they supported cell adhesion and proliferation without toxicity over 7 days of interaction. Hou et al. [10] produced microfibrous core-shell fabrics made of PCL and PGS using a wet–wet coaxial electrospinning technique. The researchers immobilised heparin on the surface of these fibres and evaluated their chemical, mechanical, and biological properties. The introduction of PGS in the composite fibres allowed for increased degradability and mechanical properties, combining structural integrity from slowly degrading PCL and fibre elasticity from PGS. The addition of PGS and heparin improved the attachment and proliferation of endothelial cells, making these core-shell fibres promising for tissue-engineering applications. Salehi et al. [11] produced aligned nanofibres composed of PGS and PCL for the purpose of cornea tissue engineering. The fibres were produced via electrospinning using varying weight ratios of PGS and PCL, resulting in diameters ranging from 300 to 550 nm. An analysis showed that an increased PGS content decreased the overall crystallinity and elastic modulus, while the surface modulus exceeded the elastic modulus and increased with the PGS content. This was thought to be due to the increasing PGS content forcing PCL into confined and cross-linked domains near the fibre surface.

Luginina et al. [12] investigated the suitability of PGS/PCL polymers for creating composite fibres incorporating bioactive glass (BG) particles. The researchers produced composite electrospun fibres with BG particles and characterised them. The addition of PGS increased the average fibre diameter, while the presence of BG particles in the composite fibres slightly broadened the diameter distribution without significantly changing the average diameter. The fibres were found to be hydrophilic, and BG particles did not affect the fibre wettability. Degradation and acellular bioactivity tests revealed the release of BG particles from the composite fibres, which could be beneficial for therapeutic applications like wound healing. However, the weak interface between BG particles and the polymeric fibres did not lead to improvements in mechanical properties. Preliminary biological tests showed promise for potential use in soft tissue engineering applications.

**Figure 1:**
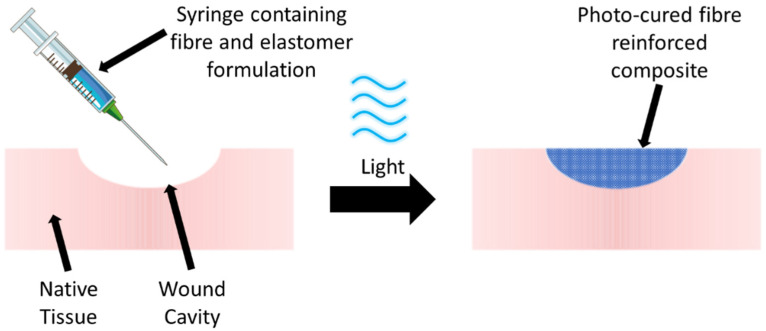
Potential clinical treatment in which a PGS-based polymer formulation containing reinforcing fibres is injected into a defect such as a wound cavity to promote repair and regeneration. Following in situ irradiation (photocuring) of the formulation at an appropriate wavelength (e.g., blue light), the fibre-reinforced PGS-based hydrogel is formed.

Despite previous reports on composite fibres made of PGS, fibre-reinforced PGS-based hydrogels are yet to be extensively explored, and such design could be leveraged to enhance the mechanical properties of the PGS phase. Other than the mechanical properties, one of the drawbacks of PGS for clinical applicability is that crosslinking the polymer requires a high temperature, which prevents any potential clinical treatment in which the polymer is injected directly into a defect site, e.g., a foot ulcer cavity, and cured in situ through an external stimulus (Figure 1). The ability to cure in situ through safe external stimuli could potentially enable less invasive key-hole clinical procedures and enable cavity defects, e.g., a foot ulcer cavity, to be more effectively filled with an injectable material to ensure full conformance to the wound surface. This surface interaction could also improve fixation, as well as the structural integrity and durability of the hydrogel following implantation. Photo-curable variants of valuable biocompatible polymers such as collagen have previously been reported [13,14,15,16] and have the potential to simplify clinical procedures.

Therefore, to extend the range of clinical applications for PGS and remove some of the current limitations, we report the synthesis of a photocurable PGS co-polymer, i.e. poly(glycerol-*co*-sebacic acid-*co*-lactic acid-*co*-polyethylene glycol) acrylate (PGSLPA), and its processing into UV-cured hydrogels and electrospun fibres. We also study the properties of PGSLPA hydrogels reinforced with electrospun nanofibres made of PGSLPA and poly(*ε*-caprolactone) (PCL) to determine the prospects for modulating mechanical properties, including the elastomeric response. The building blocks of the hydrogel composite were selected, aiming to de-risk the material’s potential use in the biological environment. Glycerol, lactic acid, PCL and Polyethylene glycol (PEG) exhibit low cytotoxicity and hold FDA approval for various applications such as dermal fillers [17]. Extensive washing and dialysis were also carried out on the final products to ensure the purification and removal of potentially toxic compounds used during either the synthesis or composite manufacture.

## 2. Materials and Methods

### 2.1. Materials

Glycerol, 4-dimethylaminopyridine and triethylamine were obtained from Fisher Scientific (Loughborough, Leicestershire, UK). Sebacic acid was obtained from Alfa Aesar (Ward Hill, MA, USA). Dichloromethane, polyethylene glycol (M_w_ 8000), stannous octanoate, p-toluenesulfonic acid monohydrate, lactic acid (85%), polycaprolactone (M_w_ 80,000), 1,1,1,3,3,3-hexafluoro-2-propanol (HFIP), polyethylene glycol diacrylate (M_w_ 8000) (PEGDA_8000_), 2-hydroxy-4′-(2-hydroxyethoxy)-2-methylpropiophenone (Irgacure 2959) and anhydrous magnesium sulphate (MgSO_4_) were obtained from Sigma Aldrich (Burlington, MA, USA).

### 2.2. Synthesis of Poly(glycerol sebacate-co-lactic acid-co-polyethylene glycol) (PGSLP)

Poly(glycerol sebacate-*co*-lactic acid-*co*-polyethylene glycol) (PGSLP) was synthesised as a starting material by adapting the method reported by Jia et al. [18]. First, a round bottom flask was charged with sebacic acid (11.0 g, 54.35 mmol), lactic acid (14.67 g, 163.04 mmol) and PEG (M_w_ 8000, 10.87 g, 1.36 mmol). This mass of lactic acid refers to the mass of 85% solution, taking into account the presence of water. The vessel was flushed with nitrogen, magnetically stirred at 350 rpm and heated to 120 °C. Once the temperature reached 120 °C, vacuum pressure (0.001 bar) was applied and stirring was continued for 4 h. Stannous octanoate, Sn(Oct)2 (1.1 g, 2.72 mmol) and p-toluenesulfonic acid monohydrate TSA·H_2_O (0.52 g, 2.72 mmol) were added, under vacuum (0.001 bar), and the temperature was increased to 180 °C. The reaction was then continued for 24 h. Glycerol (5.0 g, 54.35 mmol) was then added, and the reaction continued for a further 8 h under reduced vacuum pressure (5 × 10^−4^ bar). The material produced was a clear viscous fluid. The material was dissolved in DCM, water was removed with magnesium sulphate (5 g) and the solvent was removed via rotary evaporation. A viscous brown fluid was obtained, which was left for 24 h in a vacuum oven at 40 °C.

### 2.3. Synthesis of Photo-Functional Poly(glycerol sebacate-co-lactic acid-co-polyethylene glycol) Acrylate (PGSLPA)

PGSLP (35.0 g) and DMAP (1.66 g, 13.59 mmol) were added to a flame dried round bottom flask, and the flask was sealed and flushed with N_2_. Anhydrous dichloromethane (150 mL) was added to the flask and the magnetic stirrer was turned on at 350 rpm; the flask was then cooled in an ice bath to 0 °C. Acryloyl chloride (24.46 g, 271.74 mmol) and triethylamine (27.45 g, 271.74 mmol) were simultaneously added dropwise using dropping funnels, and the reaction was left stirring for 24 h.

The reaction mixture was poured in ethyl acetate to facilitate the precipitation of the polymer; however, no precipitate formation occurred. The mixture was placed on a rotary evaporator to remove all of the solvent, whereupon the obtained polymer was dissolved in dichloromethane and poured into n-pentane, yielding a polymer at the bottom of the flask. The solvent mixture was decanted, and the polymer was dissolved in dichloromethane and placed on a rotary evaporator. The mixture was dissolved in deionised H_2_O and dialysed over a period of 3 days with regular water changes. The polymer solution was then dried on a rotary evaporator, yielding a viscous brown fluid.

### 2.4. Preparation of Nonwovens from PGSLP and PGSLPA

Nanofibres of PGSLP and PGSLPA products were electrospun in the presence of PCL as a fibre-forming carrier polymer to accomplish a suitable degree of chain entanglement for homogeneous fibre formation. A solution of 5 wt % PGSLP (or PGSLPA) and 5 wt % PCL (M_w_ 80,000) was prepared in HFIP, transferred to a glass syringe equipped with a 18G needle tip and loaded onto a syringe pump. A charged plate with a 20 cm distance from the needle tip was secured in place, whereby an electrostatic voltage and syringe flow rate were varied in the range of 20–25 kV and 0.25–1.5 mL h^−1^, respectively, to accomplish homogeneous electrospun fibres. Samples were also produced from electrospinning solutions loaded with photoinitiator (0.2 wt % DMPA).

### 2.5. Scanning Electron Microscopy

SEM micrographs were collected on a Jeol JSM-6610LV (JEOL, Welwyn Garden City, UK) scanning electron microscope (JEOL, Welwyn Garden City, UK) employing a 10 mm focusing lens. Micrographs were collected at accelerating voltages from 15 to 30 kV and with magnification from 250 to 10,000. Different accelerating voltages were used to enhance the resolution of the nanofibres present in the nonwoven web. Magnification was changed to allow for accurate fibre diameter and porosity measurements. Samples were prepared by cutting to 10 × 10 mm, and a sputter coating was employed to produce high-quality micrographs. The SEM micrographs were analysed using ImageJ (version 1.51w), and the average fibre diameter was determined by measuring the fibre diameters of 50 individual fibres selected at random using the reference scale bar in the SEM micrograph.

### 2.6. Preparation of Photo-Curable Fibre-Reinforced PGSLPA Hydrogels

In the fibre-free configuration, a solution of 26 wt % PGSLPA, 10 wt % PEGDA (M_w_ 8000), and 1 wt % Irgacure 2959 was prepared in deionised water in the dark at 50 °C under magnetic stirring at 200 rpm. A Chromato-Vue C-71 (λ: 365 nm) was used to cure the resulting polymer solution and obtain the gels.

**Figure 2:**
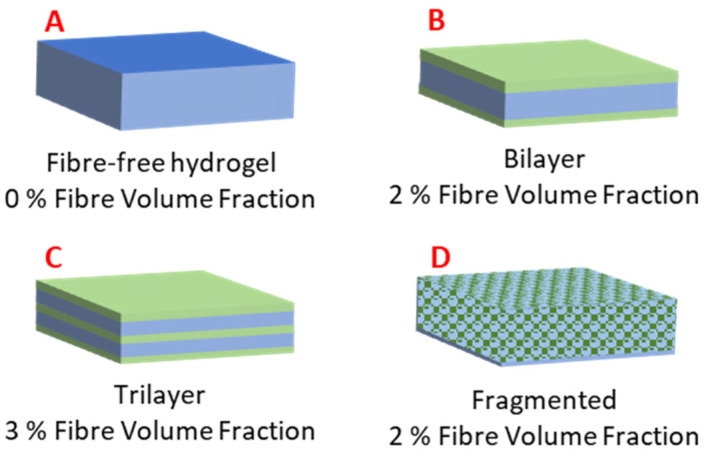
Hydrogel and electrospun nonwoven fibre-reinforced structural configurations: (**A**) Fibre-free hydrogel of PGSLPA. (**B**) Bilayer arrangement, with the nonwoven layer coated on both sides by the hydrogel. (**C**) Trilayer arrangement via sequentially layered and nonwoven hydrogel. (**D**) Hydrogel reinforced by fragmented nonwoven fibre.

The hydrogel-forming solution and respective PGSLPA/PCL nanofibrous webs were layered into a mould in three different composite configurations, as reported in Figure 2. For the fragmented nanofibre-reinforced composite, the electrospun nanofibrous web was cut into 2 × 1 mm samples and dispersed throughout the hydrogel-forming solution. The moulds were UV-irradiated for 2 h to promote photocuring.

Figure 2 shows the schematic structural arrangements of the hydrogel and nanofibre components, with the associated volume fractions of the hydrogel (matrix) and nonwoven (reinforcement) phases.

### 2.7. Mechanical Testing

Mechanical testing was carried out for the composite constituents, i.e., hydrogel and nanofibre fabric, as well for all composite configurations, in either tension or compression modes, as reported below.

#### 2.7.1. Tensile Testing of Nonwoven Fabrics

The uniaxial tensile testing of the nonwovens was conducted (James Heal, Titan Universal Strength Tester, Halifax, UK), operating with a 100 N load cell. Jaw separation was calibrated to 30 mm and checked manually using Preciva FRDM730002 Verniers (Preciva, Portland, OR, USA), with testing performed at an extension rate of 1 mm/min. Specimens were attached to cardboard templates using double-sided adhesive tape as anchor points; the effective testing length of the samples was 30 × 25 mm. Once clamped in the jaws, the collection plate was cut from the sample. Sample measurements were carried out in triplicate, and the samples were conditioned at 20 °C and 65% relative humidity for 24 h prior to testing.

#### 2.7.2. Compression Testing of Hydrogel and Nonwoven Reinforced Samples

Compression tests were conducted to sample failure, and the stress–strain response was recorded. Photocured samples of PGSLP-based fibres and hydrogels were cut to 4 × 15 × 15 mm prior to testing. An Instron 3365 Universal Tester (Instron, High Wycombe, UK) with a 500 N load cell in compression mode was used, at a strain rate of 1 mm/min and a distance of 2.5 mm. The elastic modulus of the nonwoven reinforced samples was calculated between the strain rates 0.1 and 0.6.

### 2.8. Degradation Tests

To simulate physiological conditions, electrospun samples (n = 3) were individually incubated in a phosphate buffered solution (PBS, 10 mM, pH 7.4, 25 °C) [19] for up to 8 weeks, prior to the determination of any mass loss. DMPA photoinitiator-doped samples were also tested before and after curing to determine the change in the degradation behaviour due to the introduction of covalent crosslinks at the molecular scale. The samples were cut to equal dimensions (10 × 30 mm), and the initial mass of the dry samples was measured and then re-measured at 1-week intervals to quantify the percent residual mass. All mass measurements were obtained using oven-dried samples (dried at 50 °C for 24 h, in a Binder ED56 Series static oven, BINDER, Tuttlingen, Germany). The results were linearly fitted to assess erosion-driven degradability. The fitting of a linear model using OriginPro (version 8.5) was used to determine the fitting equations and the coefficient of determination (R^2^) for each degradation profile, allowing for an understanding of the relationship between the variables time and mass.

## 3. Results and Discussion

### 3.1. Synthesis of PGSLP and PGSLPA

**Figure 3:**
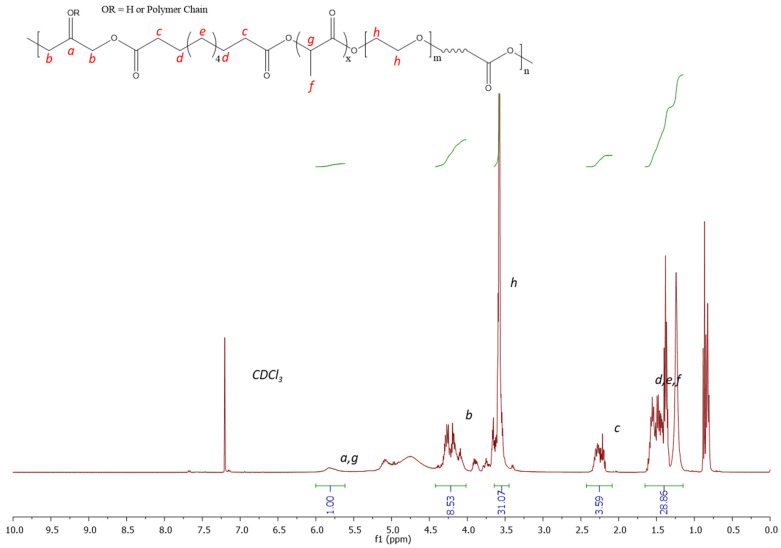
^1^H NMR 300 MHz spectrum (CDCl_3_) of PGSLP.

The synthesis of PGSLP followed a similar methodology to that described by Jia et al. [18]. The polymer was found to be soluble in deionised water with stirring. ^1^H NMR analysis (Figure 3) was performed in CDCl_3_ using a Bruker Avance III HD 400 MHz spectrometer, and the correct target structure was obtained following synthesis. Peaks at 5.1–4.9 ppm correspond to methylene protons from sebacic acid and lactic acid. Peaks at 2.25 ppm were assigned to methylene proton from sebacic acid, closest to the carboxylic acid terminal groups. The large peak at 3.55 ppm corresponds to the protons present in the PEG block. Peaks at 4–4.4 ppm correspond to methylene protons in glycerol. Peaks at 4.9–5.1 ppm were observed due to protons in glycerol and lactic acid. The polymer was further purified by dialysis, leading to a yield of 90%.

Normally, PGSLP is cured by a high-temperature treatment, which limits its clinical applicability in cases where curing may need to be carried out in situ. The development of a photocurable derivative was therefore targeted to enable a crosslinked network formation at room temperature.

Acryloyl chloride was used to graft alkene functionality—specifically, acrylate functional groups—to the free hydroxyl groups present within the polymer’s structure. Introducing a diacrylate crosslinker, such as PEG diacrylate with a suitable photoinitiator, allows for covalent bonds to be formed between the polymer chains. This bond formation allows for a gel structure to form in suitable solvents, which can then be reinforced or doped with suitable fillers.

**Figure 4:**
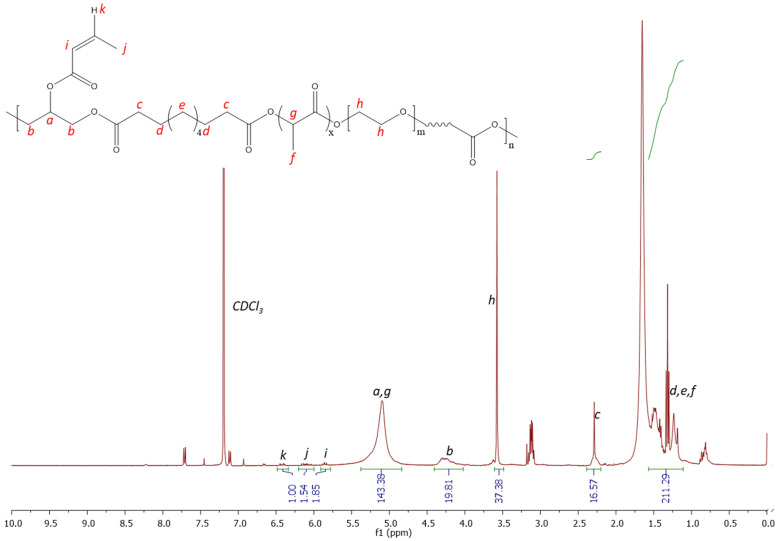
^1^H NMR spectrum 300 MHz (CDCL_3_) of PGSLPA.

Figure 4 shows the ^1^H NMR spectrum, confirming the successful grafting of acrylate functionality to the polymer structure. A similar peak structure is observed with the unmodified polymer; however, additional peaks are observed at 6.4, 6.1 and 5.6 ppm, which correspond to the successfully grafted photocuring functionality. The protons present in the alkene group (labelled *i*, *j* and *k*) are clearly observed within the spectrum, corresponding to comparable integration values, as expected. The solubility testing of the material by immersing it in deionised water showed the material to be readily dissolved with stirring, such that it could be an appealing candidate for gel formation in situ following injection directly into a wound site and photocuring.

### 3.2. Dimensional, Tensile and Degradation Properties of Electrospun PGSLP and PGLSPA Nonwovens

**Figure 5:**
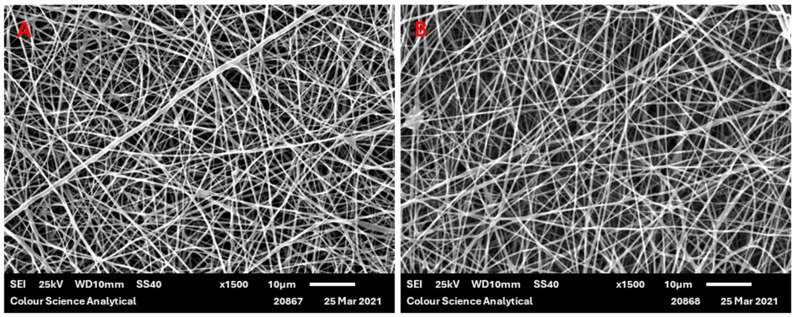
SEM micrograph of (**A**) electrospun 50:50 *w*/*w* PGSLP/PCL fibres and (**B**) electrospun 50:50 *w*/*w* PGSLPA/PCL fibres (scale bar = 10 microns).

PGSLP and PGSLPA were successfully electrospun using PCL as a carrier material in 50:50 *w*/*w* proportions. This mixing ratio ensured that no electrospraying occurred and that the electrospinning process was stable. While the commixing of these materials was expected to negatively influence the resultant elastic properties of the PGSLP and PGSLPA, polymer blending was identified as a prerequisite for the successful electrospinning of self-supporting nonwovens that could subsequently be incorporated into reinforced hydrogel constructs. Electrospun samples were also produced, containing a photoinitiator (DMPA) to permit subsequent photocuring in the composite structure, avoiding post-spinning incubation in a photoinitiator-supplemented solution [20,21]. Figure 5 shows typical examples of SEM micrographs of the produced electrospun webs, showing relatively smooth PGSLP/PCL and PGSLPA/PCL fibre morphologies. Table 1 reports the mean fibre diameters of the produced electrospun nonwovens, which were all in the range of 280 to 290 nm, with comparable standard deviation values.

The sufficient polymer chain entanglement, polymer concentration and solvent distribution over the entangled polymer molecules enabled the production of smooth, uniform nanofibres. Qian et al. [22] fabricated electrospun PCL and PCL/Chitosan-Gelatin nanofibrous mats and showed that the morphology of the PCL nanofibres was smooth, with interconnections between the fibres. This morphology was lost when blending with chitosan-gelatin, with the fibres losing this interconnection, likely due to an increase in conductivity from the blended solutions from the polar groups associated with chitosan and gelatin—specifically, the amine and carboxylic acid functionality. Luginina et al. [12] observed an increase in the average fibre diameter when mixing PCL with PGS, likely due to the total increase in the volume of the polymer content in the solution. The average fibre diameter values reported in this work are lower than those reported in the literature for PCL/PGS blends, where the fibre diameters vary in the range of 550 to 4700 nm [11,23,24,25]. This could be due to differences in the molecular weights of the PGSLP and PGSLPA produced in this work compared to those of previously reported PCL/polymer blends and the associated impact on the spinning solution viscosity, which will affect as-produced fibre diameters. Differences in the concentration of the spinning solution will also influence the fibre diameter. The solvent choice and the associated volatility, polarity and surface tension are further factors affecting the electrospinning process. Ultimately, the precise electrospinning process parameters and environment in which the fibres are produced will markedly influence the resultant mean fibre diameter.

The stress–strain curves of electrospun materials were collected on a James Heal Titan Universal Strength Tester. The stress–strain curves of the composite and gel materials were collected on an Instron 3365 Universal Tester.

**Figure 6:**
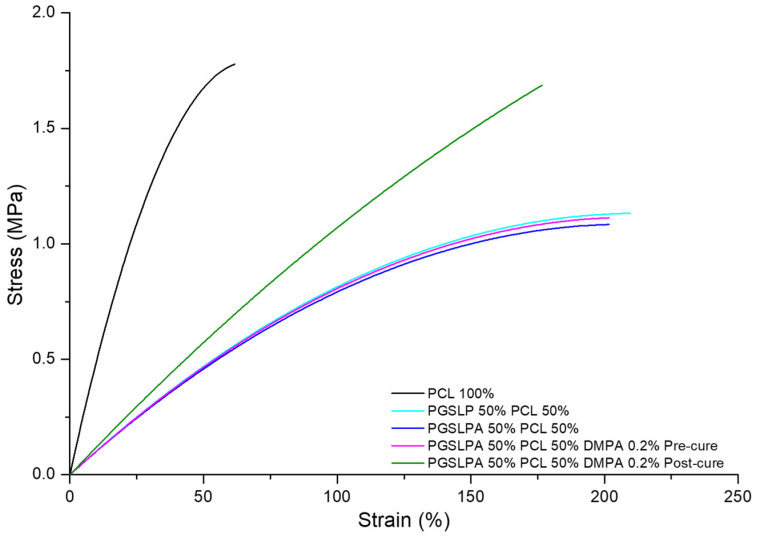
Dry state tensile stress–strain curves of the PCL control, as well as DMPA-free and DMPA-doped PGSLPA nonwovens before and after UV irradiation.

The corresponding stress–strain curves for all PGSLP and PGSLPA electrospun nonwovens are shown in Figure 6, where the measurements of a PCL-based electrospun fabric produced under identical spinning conditions are included as the control. PGSLP- and PGSLPA-containing nonwovens produced an increased toughness in their stress–strain responses compared to the 100% PCL sample (Figure 6). This observation can be explained in terms of composite mechanics and the improved stress distribution due to the elastomer distributing the strain more efficiently, reducing localised stress concentrations that may occur in a purely rigid material. The stiffness of the PCL component, when combined with the compliance (flexibility) of the PGSLP and PGSLPA elastomers, leads to an overall reduction in the effective stiffness of the composite. Modification of the molecular structure to introduce photocuring functionality resulted in no significant change in the strain response (*p* < 0.05). Electrospun PGSLPA fabrics doped with a 0.2% DMPA photoinitiator displayed no significant change (*p* < 0.05) as compared to undoped electrospun fabric, which may be attributed to the lack of covalent crosslinks and the minimal effect of secondary interactions between grafted residues. On the other hand, UV irradiation revealed a marked change in the stress–strain response, with the fabric displaying a linear stress–strain curve, as a result of covalent crosslinks introduced between PGSLPA polymer chains within the electrospun nanofibres (Figure 6).

**Figure 7:**
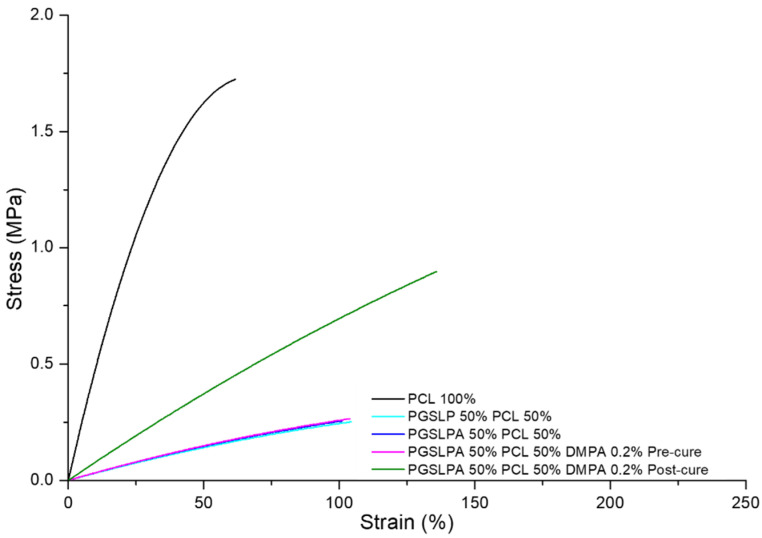
Wet state tensile stress–strain curves of the PCL control, as well as DMPA-free and DMPA-doped PGSLPA nonwovens before and after UV irradiation.

To reflect physiological conditions, wet tensile properties were also measured (Figure 7), and a marked difference between wet and dry conditions was immediately apparent for the PGSLP and PGSLPA samples.

Other than PCL, the breaking stress and strain of all the hydrated electrospun nonwovens (Figure 7) were lower than those in their dry state (Figure 6), which is a consequence of the water-induced swelling due to the hydrophilicity of the PGSLPA phase. The photo-curing of PGSLPA reduced the overall decrease in the breaking stress (47%) and breaking strain (23%) as compared to the larger decreases prior to photo-curing. This supports the synthesis of UV-induced covalent crosslinks between fibre-forming polymer chains, leading to fibres with an increased tensile strength and increased resistance to mechanical deformation.

**Figure 8:**
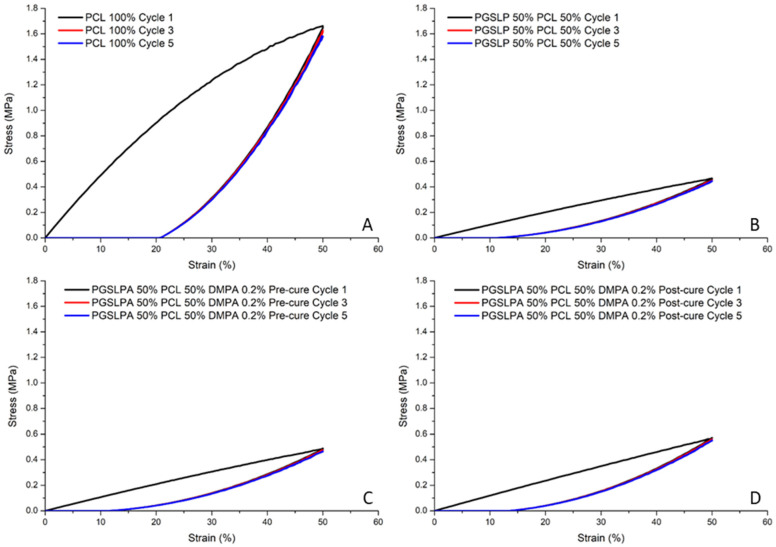
Cyclic tensile stress–strain curves (cycles one, three and five) for: (**A**) 100% PCL nanofibre fabrics; (**B**) 50% PGSLP/PCL nanofibre fabrics; (**C**) uncured 50% PGSLPA/PCL DMPA photoinitiator-doped nanofibre fabrics and (**D**) cured 50% PGSLPA/PCL DMPA photoinitiator-doped nanofibre fabrics.

Cyclical strain experiments (samples loaded to 50% strain) were carried out to assess the elastic response and revealed no marked hysteresis in the PGSLP and PGSLPA electrospun nonwovens (Figure 8B,C), most likely because of the semi-crystalline nature of the PCL present in the fibres [26]. After the initial loading (cycle 1), the equilibrated stress–strain response for subsequent cycles closely followed that of the relaxation phase of cycle 1. The mixing of semi-crystalline and amorphous polymers can result in stress concentrations at the interface between the two phases, resulting in poor hysteresis. Herein, the energy dissipated for cycle 1 is 30.4, 5.5, 5.7, and 6.6 for the materials in Figure 8A,B,C and D, respectively. The optimisation of the blend composition and the introduction of compatibilisers can enhance the interfacial adhesion and improve the hysteresis response.

Figure 8C,D again reveals increased breaking stress (post photocuring), whereby the same trend of equilibration after the initial loading cycle is observed, with subsequent loading and relaxation phases following the relaxation phase of cycle 1.

**Figure 9:**
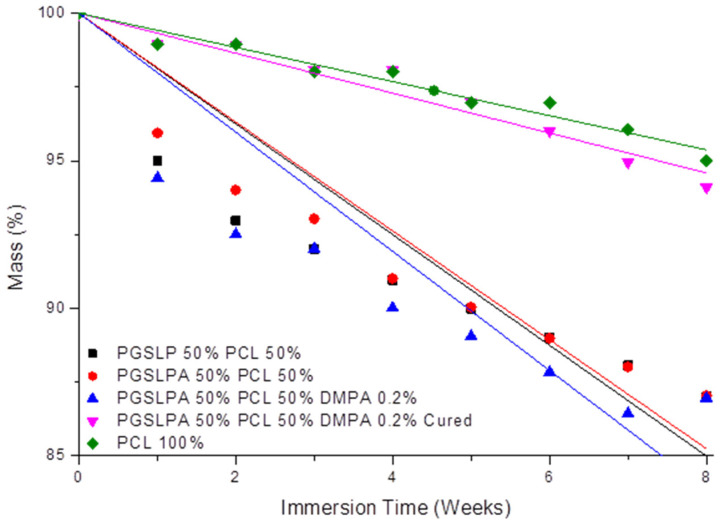
Degradation profile of PGSLP and photoinitiator-doped PGSLPA-based nonwovens before and after photocuring following long-term immersion in PBS buffer solution. The 100% PCL electrospun nonwoven sample is included as a reference. Black: PGSLP 50%, Red: PGSLPA 50% PCL 50%, Blue: PGSLPA 50% PCL 50% containing 0.2% DMPA photoinitiator, Magenta: PGSLPA 50% PCL 50% containing 0.2% DMPA photoinitiator and UV-Cured, Green: PCL 100%.

Following the characterisation of the tensile behaviour, the biodegradability of the electrospun samples was subsequently investigated. Figure 9 shows the mass loss profiles of the nanofibre fabric samples after immersion in PBS, whereby all uncured PGSLP and PGSLPA samples proved to degrade at a higher rate than the 100% PCL reference sample. Before curing, the presence of a photoinitiator did not substantially affect the mass loss, as expected given the absence of covalent crosslinks between fibre-forming polymer chains. However, after the photocuring of the photoinitiator-doped PGSLPA fabrics, the sample mass of PGSLPA was retained at a sharply increased level comparable to that of 100% PCL, with only about 5% mass loss after 8 weeks. In clinical practice, non-toxic compounds resulting from the breakdown of PGSLP and PGSLPA are easily eliminated from the body. With respect to the application as chronic wound pad materials, this polymer contrasts with currently used silicone injections, where the mechanically degraded material can migrate through the body [27]. On the other hand, PGSLP is composed of chemicals used in food applications and, in some cases, is already present in the body (lactic acid), which is intended to minimise the potential toxicity issues of the degradation products. However, the post-synthesis modification to yield PGSLPA, which has an acrylate functional group, would require detailed toxicology studies to be carried out in future work.

The degradation profiles (Figure 9 and Table 2) suggest that the nanofibre fabric samples undergo surface erosion, as there is a controlled mass loss with time. The mass loss rate decreases over time, indicating that surface erosion causes a reduction in the fibre diameter, and as the fibre diameter decreases, the surface area of the fibre therefore reduces such that less surface erosion can take place. A controlled mass loss is desirable in biomedical implants and injectable formulations, as it allows for the retention of mechanical properties as the material is removed by the body and replaced with natural tissue. Another added benefit of a controlled degradation profile is the possibility of couple degradation with the release of a therapeutic agent. The incorporation of therapeutic molecules within the structure could provide an avenue for improved clinical outcomes. PCL is one such material that has previously been investigated for its use as a long-term drug delivery vehicle [28]. The loading of PCL-based fibres with self-assembling peptides has been reported to generate bioactive electrospun meshes with a spider-net nanofibre architecture, due to the renaturation of hydrogen bonds following solvent removal [29]. Likewise, PCL-based, photosensitiser-encapsulated electrospun meshes revealed a significantly reduced fibre diameter and significantly increased tensile modulus [30,31], a consequence of the impact of loading on both the electrospinning solution viscosity and polymer crystallinity. Further studies should therefore be carried out to investigate the effect of drug loading on either the mechanical properties or the microstructure of the presented hydrogel composite.

### 3.3. Compressive Strength of Nonwoven Reinforced PGSLPA Hydrogels

**Figure 10:**
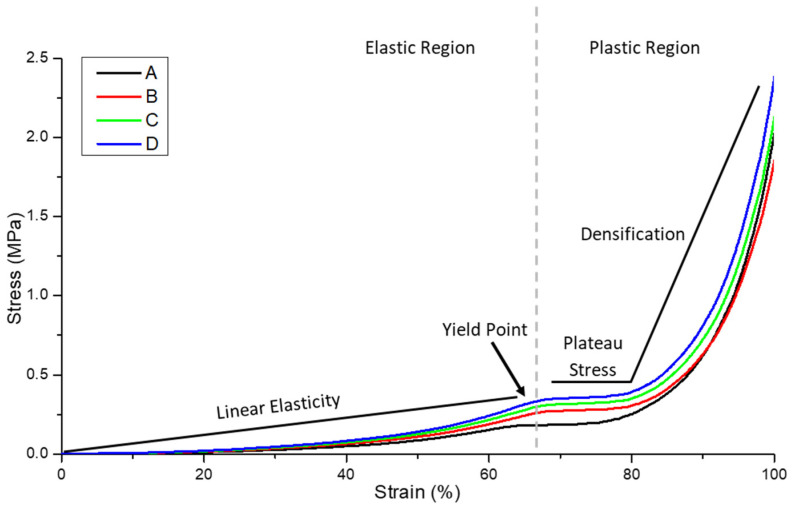
Compression stress–strain curve for the hydrogel and nanofibre-reinforced PGSLPA hydrogels. (A) 100% PGSLPA hydrogel with no reinforcement. (B) Bilayer nonwoven reinforced format. (C) Trilayer nonwoven reinforced format (D) Segmented nonwoven reinforced format. The grey dotted line indicates the transition from elastic to plastic deformation.

Compression to failure testing enabled a complete picture of the strain behaviour, indicating how the bulk material would likely perform in a soft tissue repair application. In reality, biomaterials replacing soft tissues are not always required to undergo compression to failure in situ but rather undergo cyclic compression in the linear, elastic portion of the stress–strain curve. Figure 10 illustrates the ductile stress-strain behaviour of the hydrogel-nonwoven reinforced structural formats (Figure 2B–D) and the non-reinforced hydrogel (Figure 2A). In all experiments, the nonwoven used to reinforce the hydrogel was the PGSLPA/PCL electrospun sample. Despite the very low fibre volume fraction of only ~2–3% (Figure 2), nanofibre reinforcement increased the maximum stress and modulus of both the PGSLP and PGSLPA hydrogels compared to the non-reinforced samples (Table 3).

The structural format of the hydrogel-nanofibre composite also appeared to influence the compression stress–strain response. In Figure 10, the highest stress was associated with the segmented nonwoven reinforced sample (Figure 2D). For example, at 60% strain, the stress of this sample was 59%, 29% and 12% greater than that of an unreinforced hydrogel (Figure 2A), bilayer (Figure 2B) and trilayer sample (Figure 2C), respectively.

**Figure 11:**
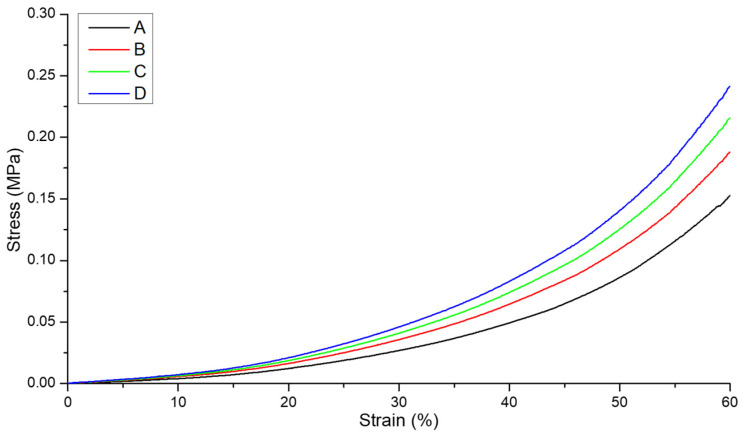
Initial linear elasticity portion of the compression stress–strain curve for the hydrogel and composite constructions. (A) 100% PGSLPA hydrogel with no reinforcement. (B) Bilayer nonwoven reinforced format. (C) Trilayer nonwoven reinforced format (D) Segmented nonwoven reinforced format.

Figure 11 shows the linear portion of the stress–strain curve profile for all four compositions, i.e., before the yield point, and this was considered for fatigue testing as well for calculating the elastic modulus (Table 3).

The elastic modulus of the segmented nonwoven reinforced sample (format D) was significantly higher (*p* < 0.05) than that of the unreinforced (format A) and bilayer (format B) constructions, at strain rates of 0.1–0.6, with the largest difference being between structural format types A and D.

**Figure 12:**
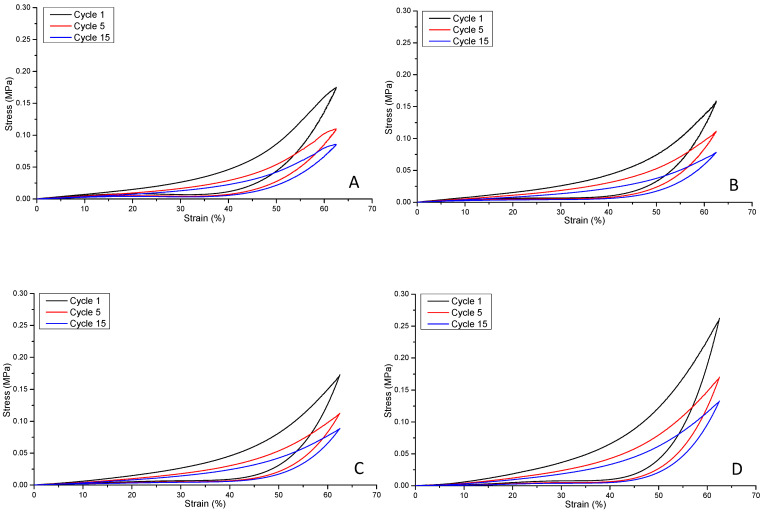
Compression fatigue stress–strain curves for: (**A**) unreinforced composite, A; (**B**) bilayer reinforced composite, B; (**C**) trilayer composite, C; (**D**) segmented electrospun nonwoven reinforced composite, D.

Figure 12 illustrates the compression fatigue stress–strain curve (hysteresis) responses of hydrogel non-reinforced and reinforced structural formats A, B, C and D, respectively. The hysteresis for the non-reinforced and reinforced PGSLPA hydrogels reveals clear elastomeric behaviour, with the deformation energy being absorbed, and samples returning to their original dimensions, as evidenced by the elastic recovery to 0% strain upon unloading. Although a similar hysteresis response was observed for all samples, including the hysteresis response observed at 50% maximum stress, at cycle 15, some differences were discernible. Cycle 1 showed an energy dissipation of 0.047, 0.041, 0.043 and 0.061, cycle 5 showed an energy dissipation of 0.029, 0.029, 0.028 and 0.040 and cycle 15 showed an energy dissipation of 0.023, 0.021, 0.023 and 0.031 for format types A, B, C and D, respectively.

A comparison of the maximum stress at cycle one and cycle five of the samples suggests that the bilayer structure (Figure 12B) produced the smallest decrease in maximum stress (30%), compared to 37% and 35% for formats A and C and D, respectively. However, cycle 15 revealed no significant differences (*p* > 0.05) in terms of maximum stress, with decreases of 51%, 51%, 49% and 50% for formats A, B, C and D, respectively.

This behaviour is explained by the fibre orientation within the composite structure. Aligned nanofibres can be expected to impart anisotropic behaviour such that the bulk structure exhibits preferential directional strength, compared to randomly oriented nanofibres that distribute the applied strain in an isotropic manner. The fibre diameter also plays an important role in determining the total surface area available for interfacial bonding with the surrounding hydrogel, affecting the overall mechanical strength of the composite. Physical contact between the fibre and hydrogel phases and interfacial bonding are essential to ensure efficient load transfer and prevent delamination or fibre pullout. Structural formats B and D (Figure 2) possessed similar fibre volume fractions, whereas for format C, the volume fraction was slightly higher, which can lead to an improved compressive strength compared to format B. However, the randomly segmented arrangement of nanofibres in structural format D is likely to explain the highest value for compressive recovery. Further improvements to the composite structure by combining the layering of nonwoven mats with an additional randomly orientated support phase to increase the fibre volume fraction to >3% could improve mechanical stress responses still further.

## 4. Conclusions

Developing new elastomeric hydrogel materials capable of load bearing is important in enabling tissue repair and regeneration in various clinical procedures, including for chronic wound care. To extend the potential clinical value of elastomeric PGS materials, it has been shown that a photocuring variant, poly(glycerol-*co*-sebacic acid-*co*-lactic acid-*co*-polyethylene glycol) acrylate (PGSLPA), can be synthesised and formed into elastomeric hydrogels, electrospun fibres and nonwovens as well as fibre-reinforced hydrogels, without the need for a high-temperature curing step. The ability to photocure PGSLPA improves the compatibility of PGS-based materials for use in minimally invasive clinical procedures and as part of injection treatments, coupled with photocuring in situ, obviating the need for the high-temperature curing step, which is normally required. The photocuring of PGSLPA hydrogels can modulate the degradation rate as well as the bulk tensile and compression stress–strain behaviour, and elastomeric behaviour was observed in compression stress–strain evaluations. The reinforcement of PGSLPA hydrogels with a 50:50 PGSLPA/PCL nonwoven produced higher compressive stress and elastic moduli than unreinforced PGSLPA hydrogels. Sharp increases in stress values may be anticipated by increasing the fibre packing fraction beyond the 3% limit explored in this study to engineer more resilient hydrogels for clinical procedures.

## Figures and Tables

**Table 1:** Mean fibre diameters of the electrospun nonwovens.

Sample	Mean Diameter (nm)
PCL 100%	280 ± 94
PGSLP 50%/PCL 50%	289 ± 94
PGSLPA 50%/PCL 50%	282 ± 91
PGSLPA 50%/PCL 50%/DMPA 0.2%	283 ± 91
PGSA 50%/PCL 50%/DMPA 0.2%	288 ± 92

**Table 2:** Linear fitting equations related to the degradation profile of nonwovens according to a surface erosion mechanism.

Nonwoven	Fitting Equation	R^2^
PCL	−0.58x + 100	0.9999
PGSLP 50% PCL 50%	−1.88x + 100	0.9995
PGSLPA 50% PCL 50%	−1.84x + 100	0.9997
PGSLPA 50% PCL 50% DMPA 0.2%	−2.02x + 100	0.9994
PGSLPA 50% PCL 50% DMPA 0.2% Cured	−0.68x + 100	0.9999

**Table 3:** Compression strain and elastic modulus data for non-reinforced and nonwoven reinforced PGSLPA hydrogel samples.

Structural Format	Polymer Composition	Concentration (wt/vol %)	Strain	E (kPa)
A Fibre-free hydrogel	PGSLPA	25	0.1	40 ± 28
			0.2	61 ± 13
			0.3	89 ± 21
	PEGDA (M_w_ 8000)	10	0.4	123 ± 31
			0.5	172 ± 19
			0.6	254 ± 19
B Bilayer (hydrogel-nonwoven-hydrogel)	PGSLPA	25	0.1	57 ± 41
			0.2	81 ± 21
	PEGDA (M_w_ 8000)	10	0.3	119 ± 26
			0.4	161 ± 42
	PGSLPA/PCL Electrospun nonwoven (2 g/m^2^)	-	0.5	218 ± 34
			0.6	313 ± 23
C Trilayer (hydrogel-nonwoven-hydrogel-nonwoven)	PGSLPA	25	0.1	64 ± 44
			0.2	93 ± 20
	PEGDA (M_w_ 8000)	10	0.3	136 ± 28
			0.4	184 ± 47
	PGSLPA/PCL Electrospun nonwoven web (2 g/m^2^)	-	0.5	250 ± 37
			0.6	359 ± 12
D Fragmented (nonwoven reinforced hydrogel)	PGSLPA	25	0.1	73 ± 53
			0.2	104 ± 28
	PEGDA (M_w_ 8000)	10	0.3	153 ± 36
			0.4	207 ± 57
	PGSLPA/PCL Electrospun nonwoven web (2 g/m^2^)	5	0.5	281 ± 49
			0.6	402 ± 38

## Data Availability

Data are contained in the article.
